# Effect of Insulin-Induced Lipodystrophy on Glycemic Control among Children and Adolescents with Diabetes in Tikur Anbessa Specialized Hospital, Addis Ababa, Ethiopia

**DOI:** 10.1155/2018/4910962

**Published:** 2018-07-04

**Authors:** Afewerki Gebremeskel Tsadik, Tesfay Mehari Atey, Teshome Nedi, Bereket Fantahun, Mamo Feyissa

**Affiliations:** ^1^Department of Clinical Pharmacy, School of Pharmacy, College of Health Sciences, Mekelle University, Mekelle, Tigray, Ethiopia; ^2^Department of Pharmacology and Clinical Pharmacy, School of Pharmacy, College of Health Sciences, Addis Ababa University, Addis Ababa, Ethiopia; ^3^Department of Pediatrics, School of Medicine, St. Paul's Hospital Millennium Medical College, Addis Ababa, Ethiopia

## Abstract

**Background:**

Lipodystrophy is one of the clinical complications of insulin injection that affects insulin absorption and leads to poor glycemic control.

**Objective:**

To assess insulin-induced lipodystrophy and glycemic control.

**Methods:**

A cross sectional study was done on 176 diabetic children and adolescents who inject insulin for a minimum of one year. First, anthropometric and clinical characteristics of the patients were recorded in questionnaire, and then observation and palpation techniques were used in assessing lipodystrophy.

**Result:**

Out of the total 176 participants, 103 (58.5%) had insulin-induced lipodystrophy, of them 100 (97.1%) had lipohypertrophy and 3 (2.9%) had lipoatrophy. Being younger, failure to rotate the injection site every week and multiple reuse of insulin syringe had significant influence in development of insulin-induced lipohypertrophy. Lipohypertrophy in turn was associated with the use of higher dose of insulin and nonoptimal glycemic control.

**Conclusion:**

Findings of this study revealed that in spite of using recombinant human insulin, the magnitude of the lipohypertrophy still remained high. Therefore, a routine workup of insulin-injecting patients for such complication is necessary, especially in the individuals who have a nonoptimal glycemic control.

## 1. Introduction

Diabetes mellitus is a huge and growing global health problem which demands modern therapy involving greater and earlier use of intensive insulin regimens in order to achieve better control of blood glucose levels and reduce the long-term risks associated with the condition [1]. However, a study recently reported an alarming magnitude of diabetic patients having poor glycemic control in spite of their routine insulin use [2]. An obvious explanation for this condition is lacking; however, a study linked this poor glycemic control with insulin administration, storage, handling, and skin complications [3].

Lipoatrophy or local fat loss is one of the skin complications of insulin injection and is clinically characterized by visible cutaneous depression and palpable atrophy of subcutaneous fat tissue at the injection site. It may result from a local immune reaction against impurities of the insulin preparations and due to the use of purified human insulin preparations; this condition has dramatically decreased since the 1950s [4, 5]. However, lipohypertrophy has been a recognized complication of insulin therapy for many years, yet researches showed that its prevalence in insulin-injecting patients with diabetes remains greater than 50% [4, 6, 7]. Lipohypertrophy is characterized by a tumor-like swelling of fatty tissue around subcutaneous insulin injection sites [8]. Histologically, the hypertrophic adipocytes are twice as large as those from normal subcutaneous areas and contained numerous small lipid droplets. Electron microscopic analysis also revealed a minor population of small adipocytes, suggesting active differentiation or proliferation [7].

Lipohypertrophy area becomes hyposensitive. Once the patient feels pain when injecting elsewhere, but not in the lipohypertrophic area, he or she tends to continue injecting in the same site even if aware of the need to rotate sites [4]. The injection of insulin into a site of lipodystrophy may lead to erratic absorption of the insulin, with the potential for poor glycemic control and unpredictable hypoglycemia [7, 9, 10].

Factors such as insulin use time, gender, body mass index, injection site, recurrent tissue trauma from failure to rotate injection sites, and the frequency of needle reuse have been reported to be associated with the development of lipohypertrophy [6, 11, 12]. Despite factors which lead to lipohypertrophy have been identified, it is still continuing to be high. However, scanty data are available regarding lipodystrophy in Ethiopia. Therefore, this study was done to determine magnitude of lipodystrophy and identify associated factors as well as to assess the impact of lipodystrophy on glycemic control.

## 2. Methods and Patients

The study was conducted at diabetic center of Tikur Anbessa Specialized Hospital, Ethiopia using a cross sectional study design. All children and adolescent patients who used insulin for a minimum of one year and visited the center from April to July 2017 were enrolled in the study. Patients whose insulin treatment was only transient including decompensated patients with acute hyperglycemia, hospitalized patients with short-term insulin requirement, HIV patients, and patients with Cushing's syndrome were excluded from the study. Participants were included to this study based on their availability during their routine outpatient clinic visit within the study period. A total of 300 type 1 diabetic patients were screened within the predetermined period, and 176 patients who have fulfilled inclusion criteria were included using the consecutive sampling technique. Data collectors were not having knowledge of the lipodystrophy status of patients at study entry, when an assessment of their injection technique as well as examination of their injection sites was made later.

A questionnaire was developed using a relevant literature search and incorporating the recent Forum for Injection Technique (FIT) and Association of Clinical Diabetologists-Italian Diabetes Healthcare Professionals (AMD-OSDI) consensus for insulin injection technique [9, 13]. Glycated hemoglobin (HgbA1c) test is the most reliable form of diabetes diagnostic assessment, providing a good indication of glycemic control over several months [14]. HgbA1c value < 7.5% was normally accepted as an optimal level of control in children less than 19 years [14, 15]. Unlike lots of studies, in this study, glycemic control was not assessed by glycemic variability of participants since most of them were not performing self-blood glucose monitoring. Hypoglycemia was defined as the occurrence of one or more symptoms of hypoglycemia (such as poor concentration, irritability, palpitation, tiredness, sweating, strong hunger, dizziness, and tremor) and a confirmed blood glucose meter reading of less than or equal to 70 mg/dL [6]. “Frequent unexplained hypoglycemia” was defined as having one or more hypoglycemic episode per week in the absence of a definable precipitating event, such as a change in medication, diet, or activity [6].

Observation and palpation techniques were used in assessing lipodystrophy in these diabetics [6]. A thorough palpation technique (slow circular and vertical fingertip movements followed by repeated horizontal attempts on the same spot) was done. Health professionals were also advised to be gentle while touching the skin at the beginning and start to progressively increase finger pressure thereafter. They were also suggested to perform the pinch maneuver when perceiving a harder skin, to confirm their first impression by comparing the thickness of the suspected spot to that of surrounding areas. Smaller and flatter lesions were best identified by repeating all abovementioned palpation maneuvers [16, 17]. Lipodystrophy was assessed as “present” or “not present”. The presence of a noticeable or palpable lump at the injection site indicated that lipodystrophy was present. Accordingly, lipodystrophy was defined to have different grades based on morphology and pathogenesis. Grade 1 = a small protruding lipohypertrophy, grade 2 = large lipohypertrophy, and grade 3 = lipoatrophy characterized by subcutaneous fatty tissue atrophy [18].

Five experienced nurses working in diabetes clinic were recruited to extract data and to examine cases. One of them was extracting all the necessary data based on the questionnaire and the second nurse was performing the visual inspection and palpation of injection sites to detect lipodystrophy. This type of data collection technique was intentionally applied to reduce bias that would happen if a single data collector was involved in both activities.

Data was entered using EpiData® version 3.1 and analysed using SPSS® version 21. Chi-square test and bivariate logistic regression with 95% confidence level (CI) were calculated. Multivariate logistic regression was used for the adjustment of potential confounders. *p* value ≤ 0.05 was considered as statistically significant findings.

## 3. Results

### 3.1. Sociodemographic and Clinical Characteristics

Of the total 176 patients participated in this study, male to female ratio was almost equal (1 : 1.05). The mean (±standard deviation (SD)) age of the participants was 11.36 (±3.96) years and ranged from two to 18 years. Children (100, 56.8%) and adolescents (76, 43.2%) shared almost comparable proportion of the total population. Insulin injectors with the primary level of education constituted the highest percentage (85, 48.3%). Comparable proportions of parents (99, 56.3%) and patients themselves (77, 43.7%) were in charge of injecting insulin daily. About three-fourths (130, 73.9%) of the patients had a healthy weight whereas only few (10, 5.7%) participants were obese based on percentile-adjusted body mass index (BMI). Approximately two-thirds (111, 63.1%) of the participants were on insulin treatment for less than five years. Almost all (173, 98.3%) patients were on combination treatment using intermediate-acting insulin and regular insulin, and frequency of injection was two times per day in all patients. Higher frequency (105, 59.7%) of the patients was observed on insulin dose of greater than 0.7 units/kg. Insulin syringe needle size of each patient was 8 mm.

**Figure 1:**
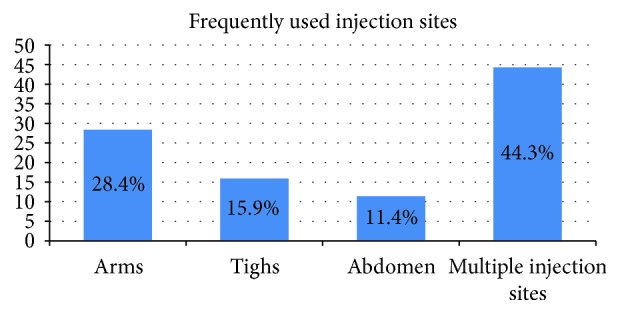
Frequently used insulin injection site among study participants.

Only few (33, 15.3%) participants were changing insulin syringe at every injection time. One in three (54, 30.7%) of the patients move or rotate the injection site every week. Above half (102, 57.9%) of the participants reported measuring with 1–3 fingers apart from the previous injections. Forty-four percent of the participants injected on multiple injection sites. Injection on arms (50, 28.4%) constituted the largest proportion followed by on thighs (28, 15.9%) and on abdomen (20, 11.4%) (Figure 1).

### 3.2. Insulin-Induced Lipodystrophy

**Figure 2:**
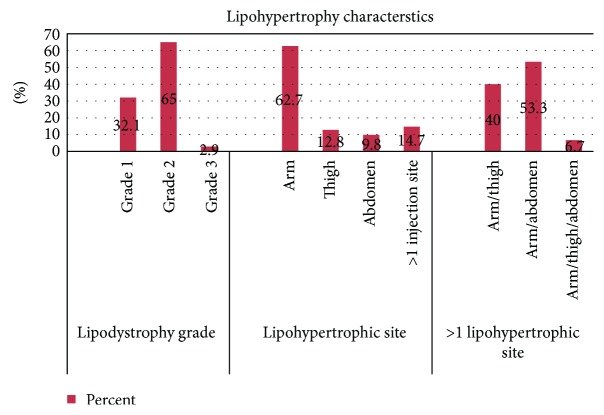
Characteristics of insulin-induced lipohypertrophy among patients with type 1 diabetes.

The prevalence of insulin-induced lipodystrophy was 58.5%, in which lipohypertrophy accounted for 97.1% of the lipodystrophy variant and the rest (2.9%) was lipoatrophy. Further, in disaggregation of lipohypertrophy by a grading scale, grade 2 lipodystrophy was the commonest type accounted for two-thirds of lipohypertrophy (67, 65%). The lipohypertrophic site was commonly observed on arms (64, 62.7%) (Figure 2). Since almost all lipodystrophies were lipohypertrophy in type, it will be used to refer to these lesions in the rest of this report.

### 3.3. Predictors of Insulin-Induced Lipohypertrophy

**Figure 3:**
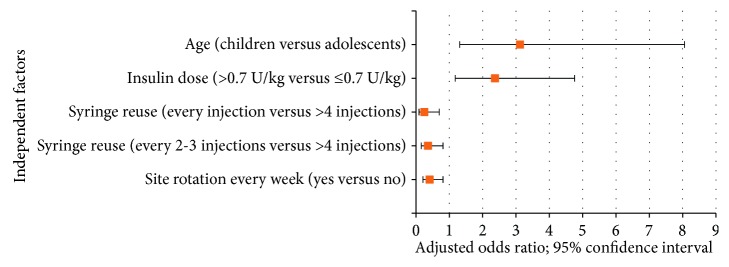
Independent factors associated with insulin-induced lipohypertrophy among type 1 diabetic patients.

Patients with lipohypertrophy and without lipohypertrophy did not differ significantly by gender, educational level of injectors, BMI, insulin use time, insulin type, space measurement on injection, and frequent unexplained hypoglycemia. However, lipohypertrophy happened significantly among children, higher insulin dose users (>0.7 U/kg), patients who failed to rotate the injection site every week, and those who practiced multiple needle reuses (*p* < 0.05) (Table 1 and Figure 3).

### 3.4. Effect of Lipohypertrophy on Glycemic Control

The presence of insulin-induced lipohypertrophy was significantly (*p* = 0.009) associated with the occurrence of nonoptimal glycemic control. The odds of having nonoptimal glycemic control were three-fold higher in patients with insulin-induced lipohypertrophy (COR = 2.943, 95% CI (1.303–6.649)) compared to those in patients without insulin-induced lipohypertrophy. However, severity of lipohypertrophy (*p* = 0.107) and site of lipohypertrophy (*p* = 0.555) were not significantly associated with the occurrence of nonoptimal glycemic control (Table 2).

## 4. Discussion

In the present study, 56.8% of the patients presented clinical evidence of lipohypertrophy, comparable with the previous similar studies done in Spain (64.4%) [6], Dublin (51%) [9], Pretoria (52%) [4], and Alexandria (54.9%) [7]; however, higher than results found from similar studies done in Saudi Arabia 47% [15] and Safari Township 15.9% [19], cohort study in Germany 47.8% [20], and an interventional study from 22 centers of seven European countries 30% [21]. This might have happened from participants' age difference and difference in insulin use duration between the studies.

Grade 2 lipodystrophy was found in 65% of the patients. This finding was not in line with the previous studies which reported that grade 1 was the commonest type of lipodystrophy [7, 15]. However, like the previous studies [6, 19], grade 3 (lipoatrophy) was still continuing to be a rare (2.9%) condition in this study too. This could be due to the introduction of high purity human recombinant insulin. Lipohypertrophy was seen to develop mostly in the arms; this might be tied to the high number of patients injected in their arms, and mostly, admonition was by parents. This result was also congruent with the reports of other studies [7, 19].

Injection site lipohypertrophy occurred almost 3 times higher among children compared to adolescents. This was also reported similarly by other studies [11, 19]. It might be due to the fact that skin thickness increases as age increases [22]; therefore, lipohypertrophy might be easily visualized and felt in children with less skin thickness than adolescents. Another plausible reason might be that parents were highly associated with occurrence of lipohypertrophy, and they were taking responsibility of injection in this class of age. Unlike our data, many studies reported the role of BMI as an effective factor in the development of the lipohypertrophy [7, 15, 19, 23]. The main reason to this dissimilarity could be the different classifications of BMI.

Development of lipohypertrophy was correlated with the use of higher insulin dose. This could be due to defective absorption of insulin by the abnormal injection sites. The same finding was also revealed by study conducted by Omar et al. [7]. Likewise, odds of nonoptimal control of blood sugar were three-fold more likely to occur among patients with lipohypertrophy compared to those patients without lipohypertrophy. Similarly, other relevant studies [15, 19, 20] have shown such findings. This finding could be attributed to the altered insulin absorption in the lipohypertrophic areas. It is vital to note that blood glucose control in the diabetes is the main aim of preventing systemic complications; therefore, patients should mandatorily stop injecting into the abnormal injection site for months to years till the lipohypertrophic areas recover to normal [22]. Even though International Diabetes Federation [24] recommends at least 3 or 4 injections/day, in this study, all patients were having 2 injections/day. Since this practice was common in both patients with lipohypertrophy and without lipohypertrophy, it might have not affected the relationship between lipodystrophy and glycemic control. One study [6] has revealed that frequent unexplained hypoglycemia was another adverse metabolic outcome of lipohypertrophy that lead to six times more commonly to occur compared to those without lipohypertrophy. But this was not reproduced by our study. The reason stated was switching injections of similar insulin dose that was being injected in the lipohypertrophic site to normal areas.

In the present study, all participants were using insulin syringes of 8 mm in length which was not in line with recommendations from Frid et al. [22], Gentile et al [3], and FIT (Forum for Injection Technique) of UK [25] and Canada [26]. Insulin syringes longer than 6 mm may result in intramuscular injection which leads to fast absorption and hypoglycemic events as well as painful injections. Participants were also observed to reuse syringes for more than one time. As a result, development of lipohypertrophy was associated with multiple usage of a single insulin syringe. This finding was also supported by other studies [15, 21]. This could be due to deformation of the needle on repeated use that it can either raise injection morbidity or, more likely, render the patient susceptible to lipohypertrophy by inducing bleeding or infection at the injection site.

Like the previous studies, injection site rotation was the main factor which was associated with the development of lipohypertrophy [6, 9, 11, 15, 21, 27]. This might be due to the repeated traumatization of the injection sites that leads to the hypertrophic lipid cells to replace the middermal collagen [15]. Therefore, guidelines recommend that patients should divide each injection site into quadrant (or halves when using thighs or buttocks) then using one quadrant per week and moving always clockwise. Injections within any quadrant or half should be spaced at least 1-2 cm from each other in order to avoid repeat tissue trauma [3, 22, 25, 26].

Detection and characterization of lipodystrophy in the diabetics were done only by observation and palpation techniques. However, it would have been better if it was supported by ultrasonography imaging since observation and palpation techniques might be affected by skin thickness and total areas of the injection sites.

## 5. Conclusions

Despite using the new recombinant human insulin, lipohypertrophy is still continuing to be high. The main factors that influenced the development of lipohypertrophy were younger age, not rotating the injection site every week, and multiple reusing of an insulin syringe. Lipohypertrophy in turn has affected patients' insulin daily dose and HgA1C to be higher. Therefore, a routine workup of insulin-injecting patients for such complication is necessary, especially in the individuals who have a nonoptimal glycemic control.

## Figures and Tables

**Table 1:** Insulin-induced lipohypertrophy by different variables among type 1 diabetic patients.

Variable	Lipohypertrophy		COR, 95% CI	AOR, 95% CI
	No (*n*, %)	Yes (*n*, %)		
Age (years)				
Children (1–12)	35 (35)	65 (65)	1.86 (1.01–3.41)^∗^	3.12 (1.31–8.06)^∗^
Adolescents (13–18)	38 (50)	38 (50)	1	1
Sex				
Male	33 (38.4)	53 (61.6)	1.28 (0.70–2.34)	1.17 (0.56–2.47)
Female	40 (44.4)	50 (55.6)	1	1
Educational level of insulin injectors				
No schooling	1 (50)	1 (50)	1.00 (0.06–17.41)	0.85 (0.04–19.0)
Primary	32 (37.6)	53 (62.4)	1.65 (0.73–3.76)	0.83 (0.29–2.36)
Secondary	24 (42.1)	33 (57.9)	1.37 (0.57–3.28)	1.05 (0.35–3.13)
Higher	16 (50)	16 (50)	1	1
Insulin injector				
Patient	37 (48.1)	40 (51.9)	0.62 (0.34–1.13)	0.66 (0.28–1.52)
Parent	36 (36.4)	63 (63.6)	1	1
BMI				
Underweight	14 (50)	14 (50)	0.67 (0.15–2.88)	0.33 (0.05–2.02)
Healthy weight	52 (40)	78 (60)	1.00 (0.27–3.71)	0.52 (0.11–2.48)
Obese	4 (40)	6 (60)	1	1
Insulin use time (yrs)				
1–5	46 (41.4)	65 (58.6)	1.01 (0.54–1.87)	0.71 (0.34–1.49)
>5	27 (41.5)	38 (58.5)	1	1
Daily insulin dose/kg				
≤0.7 U/kg	37 (52.1)	34 (47.9)	1	1
>0.7 U/kg	36 (34.3)	69 (65.7)	2.09 (1.13–3.86)^∗^	2.37 (1.18–4.76)^∗^
Frequent unexplained hypoglycemia				
Yes	27 (48.2)	29 (51.8)	1	1
No	46 (38.3)	74 (61.7)	1.49 (0.79–2.84)	1.31 (0.57–2.94)
Insulin syringe reuse				
Every injection	18 (54.4)	15 (45.5)	0.26 (0.10–0.65)^∗^	0.25 (0.09–0.70)^∗^
Every 2-3 injections	42 (47.7)	46 (52.3)	0.34 (0.16–0.71)^∗^	0.36 (0.16–0.81)^∗^
>4 injections	13 (23.6)	42 (76.4)	1	1
Site rotation every week				
Yes	31 (57.4)	23 (42.6)	0.39 (0.20–0.75)	0.41 (0.21–0.81)^∗^
No	42 (34.4)	80 (65.6)	1	1
Space measurement to inject in the same site				
Yes	48 (42.9)	64 (57.1)	0.85 (0.45–1.59)	1.02 (0.53–1.96)
No	25 (39.1)	39 (60.9)	1	1

^∗^Statistically significant: *p* ≤ 0.05. Abbreviations: BMI: body mass index; COR: crude odds ratio; AOR: adjusted odds ratio.

**Table 2:** Effect of lipohypertrophy on glycemic control for type 1 diabetic patients.

Variable	Glycemic control		COR, 95% CI	*p* value
	Optimal (*n*, %)	Nonoptimal (*n*, %)		
Lipohypertrophy				0.009^∗^
Yes	11 (10.7)	92 (89.3)	2.943 (1.303–6.649)^∗^	
No	19 (26)	54 (74)	1	
Lipohypertrophy grade				0.107
Grade 1	1 (3.0)	32 (97)	5.614 (0.687–45.878)	
Grade 2	10 (14.9)	57 (85.1)	1	
Lipohypertrophy site				0.555
Arm	5 (7.8)	59 (92.2)	2.95 (0.62–14.04)	
Thigh	2 (15.4)	11 (84.6)	1.375 (0.192–9.834)	
Abdomen	1 (10)	9 (90)	2.250 (0.200–25.369)	
More than 1 site	3 (20)	12 (80)	1	

^∗^Statistically significant: *p* ≤ 0.05, COR: crude odds ratio.

## Data Availability

The datasets supporting the conclusions of the study are available publicly in server of Addis Ababa University. Any additional data will be available on request.

## Abbreviations

AMD-OSDI: Association of Clinical Diabetologists-Italian Diabetes Healthcare Professionals
AOR: Adjusted odds ratio
BMI: Body mass index
CI: Confidence interval
COR: Crude odds ratio
FIT: Forum for Injection Technique
HbA1C: Glycated hemoglobin
HIV: Human immunodeficiency virus
mg/dL: Milligram per deciliter
OR: Odds ratio
SD: Standard deviation.
